# Efficient estimation of population variance of a sensitive variable using a new scrambling response model

**DOI:** 10.1038/s41598-023-45427-2

**Published:** 2023-11-14

**Authors:** Iram Saleem, Aamir Sanaullah, Laila A. Al-Essa, Shakila Bashir, Aned Al Mutairi

**Affiliations:** 1 Department of Statistics, Forman Christian College (Chartered University), Lahore, Pakistan; 2 Department of Statistics, COMSATS University Islamabad, Lahore Campus, Lahore, Pakistan; 3 Department of Mathematical Sciences, College of Science, Princess Nourah bint Abdulrahman University, P.O.Box 84428, Riyadh, 11671 Saudi Arabia

**Keywords:** Applied mathematics, Computational science, Scientific data, Statistics

## Abstract

This study introduces a pioneering scrambling response model tailored for handling sensitive variables. Subsequently, a generalized estimator for variance estimation, relying on two auxiliary information sources, is developed following this novel model. Analytical expressions for bias, mean square error, and minimum mean square error are meticulously derived up to the first order of approximation, shedding light on the estimator’s statistical performance. Comprehensive simulation experiments and empirical analysis unveil compelling results. The proposed generalized estimator, operating under both scrambling response models, consistently exhibits minimal mean square error, surpassing existing estimation techniques. Furthermore, this study evaluates the level of privacy protection afforded to respondents using this model, employing a robust framework of simulations and empirical studies.

## Introduction

Information regarding complex characteristics for example family income, induced abortions, criminal activities, etc. may cause refusal bias, response bias, or both. The randomized response technique (RRT) was introduced by Warner^1^ to overcome such a difficult situation where the response is qualitative. In RRT, the information attained ensures the privacy of the respondent. This work was extended to quantitative response models with scrambling responses. Pollock and Bek^2^ presented the theory of additive and multiplicative scrambling randomized response (SRR) technique. On the choice of scrambling mechanism, many researchers made an effort to develop models such as Himmelfarb and Edgell^3^, Eichhron and Hayre^4^, Gupta et al.^5^, Diana and Perri^6^, Hussain and Khan^7^, Zaman et al.^8^, and Azeem^9^. Besides, developments by different survey researchers are made to the estimation stage using additive models. Sousa et al.^10^ first presented ratio-type estimators estimating the population mean of the complex study variable using the non-delicate auxiliary variable. Additionally, Koyuncu et al.^11^, Gupta et al.^12^, Saleem et al.^13^, Shahzad et al.^14^, Sanaullah et al.^15,16^, Saleem and Sanaullah^17^, Khalid et al.^18^ and Juarez-Moreno et al.^19^ presented various estimators for the population mean of the complex variable using different RRT models.

In human regular life, variation remains existent everywhere. Naturally, not the two individuals or things are identical. In all fields, we necessitate estimating the population variance, such as the climate factors from place to place, the degree of blood pressure, etc. The medical researcher needs a suitable understanding of the level of variation of a particular HIV treatment dose curing or affecting from person to person to be able to plan whether to reduce or change the treatment for a particular person. Practically, several situations can be seen where the estimation of population variance can be observed for complex issues. In survey sampling, the auxiliary variable is used to intensify the precision of population variance estimators at the stage of estimation. The work on the estimation of population variance for the non-sensitive variable of interest was done by countless statisticians such as Gupta and Shabbir^20^, Asghar et al.^21^, Sanaullah et al.^22^, Niaz et al.^23^ and Zaman and Bulut^24^. Singh et al.^25^ first introduced a new estimator to estimate the population variance of the sensitive variable of interest centered on a multiplicative scrambled response model using auxiliary information. They presented different procedures for estimating variance using Das and Tripathi^26^ and Isaki^27^ estimators. Later, Gupta et al.^28^ presented three variance estimators under Diana and Perri’s^6^ RRT model using two scrambling variables. Aloraini et al.^29^ proposed some separate and combined variance estimators using stratified sampling following the strategy presented by Gupta et al.^28^.

The present study follows the methodology of Gupta et al.^28^ and suggested a new generalized exponential estimator, to estimate the variance of the finite population which is complex in nature. The jargon of the bias and the mean square error of the proposed estimator originated up to the first order of approximation. The outline of the article is organized as follows: In “Sampling strategy for scrambled response model” section the sampling strategy for the scrambled response model presented by Diana and Perri^6^ is discussed. “The proposed estimator and its class of estimators” section, displays the proposed generalized estimator for two auxiliary variables under the existing model along with the expressions of bias and MSE. In “The proposed RRT model and estimator-II” section, we also propose a generalized randomized response model. The unbiased variance estimator, ratio estimator, and proposed generalized estimator are modified under the proposed model in the same section. The privacy protection measure for the models are discussed in “Privacy levels” section. To support the proposed methodology a simulation study is presented in “An application of the proposed model” section and some concluding interpretations are given in “Simulation study” section.

## Sampling strategy for scrambled response model

Let a simple random sample done without replacement (SRSWOR) of size *n* be drawn a finite population of *U* = {*U*_1_, *U*_2_,…, *U*_*N*_}. Let *Y* be a true response of sensitive quantitative variables and *X* be the non-sensitive auxiliary variable, positively correlated to *Y*. Let ${s}_{y}^{2}=$ $\frac{\sum_{i=1}^{n}{({y}_{i }- \overline{y })}^{2}}{(n-1)}$, ${s}_{x1}^{2}=$ $\frac{\sum_{i=1}^{n}{({x}_{1i }- {\overline{x} }_{1})}^{2}}{(n-1)}$, ${s}_{x2}^{2}=$ $\frac{\sum_{i=1}^{n}{({x}_{2i }- {\overline{x} })}^{2}}{(n-1)}$, ${s}_{z}^{2}=\frac{\sum_{i=1}^{n}{({z}_{i }- \overline{z })}^{2}}{(n-1)}$, ${\sigma }_{y}^{2}=\frac{\sum_{i=1}^{N}{({Y}_{i }- \overline{Y })}^{2}}{(N-1)}$, ${\sigma }_{x1}^{2}=\frac{\sum_{i=1}^{N}{({X}_{1i }- {\overline{X} }_{1})}^{2}}{\left(N-1\right)}$, ${\sigma }_{x2}^{2}=\frac{\sum_{i=1}^{N}{({X}_{2i }- {\overline{X} }_{2})}^{2}}{\left(N-1\right)},$ ${\sigma }_{z}^{2}=\frac{\sum_{i=1}^{N}{\left({Z}_{i }- \overline{Z }\right)}^{2}}{\left(N-1\right)}$, ${\overline{X} }_{1}= \frac{\sum_{i=1}^{N}{X}_{1i }}{N}$, ${\overline{X} }_{2}= \frac{\sum_{i=1}^{N}{X}_{2i }}{N} ,$ $\overline{Y }= \frac{\sum_{i=1}^{N}{Y}_{i }}{N}$, $\overline{Z }=\frac{\sum_{i=1}^{N}{Z}_{i }}{N}$, $\overline{y }= \frac{\sum_{i=1}^{n}{y}_{i }}{n}$, ${\overline{x} }_{1}= \frac{\sum_{i=1}^{n}{x}_{1i }}{n}$, ${\overline{x} }_{2}= \frac{\sum_{i=1}^{n}{x}_{2i }}{n}, \overline{z }= \frac{\sum_{i=1}^{n}{z}_{i }}{n}$.

Let us define the following assumptions and expectations to get the bias and mean square error: ${s}_{z}^{2}={\sigma }_{z}^{2}(1+{\delta }_{z})$, ${s}_{{x}_{1}}^{2}={\sigma }_{{x}_{1}}^{2}$(1 + ${\delta }_{{X}_{1}}$), ${s}_{{x}_{2}}^{2}={\sigma }_{{x}_{2}}^{2}$(1 + ${\delta }_{{X}_{2}}$), and $\overline{z }$ = $\overline{Z }(1+{e}_{z}$), where ${\delta }_{z}$ $=\frac{{s}_{z}^{2}-{\sigma }_{z}^{2}}{{\sigma }_{z}^{2}}$, ${\delta }_{{X}_{1}}= \frac{{s}_{{x}_{1}}^{2}-{\sigma }_{{x}_{1}}^{2}}{ {\sigma }_{{x}_{1}}^{2}}$, ${\delta }_{{X}_{2}}=\frac{{s}_{{x}_{2}}^{2}-{\sigma }_{{x}_{2}}^{2}}{{\sigma }_{{x}_{2}}^{2}}$ and ${e}_{z}=\frac{\overline{z }-\overline{z} }{\overline{z} }$, such that $E\left({\delta }_{z}\right)=E\left({\delta }_{x}\right)=E\left({e}_{z}\right)=0$, $E\left({\delta }_{z}^{2}\right)=\theta ({\lambda }_{400}-1)$, $E\left({\delta }_{x1}^{2}\right)=\theta ({\lambda }_{040}-1)$, E$\left({\delta }_{x2}^{2}\right)=\theta ({\lambda }_{004}-1)$, ${E(e}_{z}^{2})=\theta {C}_{z}^{2}$, ${E(\delta }_{z}{e}_{z})=\theta {\lambda }_{300}{C}_{z}$, $E\left({\delta }_{x1}{e}_{z}\right)=\theta {\lambda }_{120}{C}_{z}$, $E\left({\delta }_{x2}{e}_{z}\right)=\theta {\lambda }_{102}{C}_{z}$, $E\left({\delta }_{x1}{\delta }_{x2}\right)=\theta ({\lambda }_{022}-1)$, $E\left({\delta }_{z}{\delta }_{x1}\right)=\theta ({\lambda }_{220}-1)$, and $E\left({\delta }_{z}{\delta }_{x2}\right)=\theta ({\lambda }_{202}-1)$.

Based on the Diana and Perri^6^ RRT model *Z* = *TY* + *S*, Gupta et al.^28^ introduced basic variance and some ratio-type estimators. The basic variance estimator is as

1 $$t_{0} = \sigma_{y}^{2} = \frac{{\sigma_{z}^{2} - \sigma_{S}^{2} - \left( {\sigma_{T}^{2} \overline{Z}^{2} } \right)}}{{\sigma_{T}^{2} + 1}}.$$

The MSE of *t*_0_ is as,

2 $$MSE\left({t}_{0}\right)= \theta \left(\frac{1}{{\left({\sigma }_{T}^{2}+1\right)}^{2}}\right)\left({\sigma }_{z}^{4}\left({\mu }_{400}-1\right)+4{\sigma }_{T}^{2}{\overline{Z} }^{2}{C}_{z}^{2}-4{\sigma }_{z}^{2}{\sigma }_{T}^{2}{\overline{Z} }^{2}{\mu }_{300}{C}_{z}\right).$$

The ratio estimators is given by,

3 $${t}_{ratio}= \frac{{s}_{z}^{2}-{\sigma }_{s}^{2}-{\sigma }_{T}^{2}\times {\overline{z} }^{2}}{{\sigma }_{T}^{2}+1}\times \left(\frac{{\sigma }_{x}^{2}}{{s}_{x}^{2}}\right).$$

The MSE of *t*_ratio_ estimator is as,

4 $$ \begin{aligned}MSE\left({t}_{ratio}\right) & = \theta \frac{1}{{\left({\sigma }_{T}^{2}+1\right)}^{2}}\left[{\sigma }_{z}^{4}\left({\mu }_{400}-1\right)-2{\sigma }_{z}^{2}{\sigma }_{x1}^{2}\left({\mu }_{220}-1\right)\left({\sigma }_{T}^{2}+1\right)+{\sigma }_{s}^{2}\left({\mu }_{040}-1\right){\left( {\sigma }_{T}^{2}+1\right)}^{2}\right]\\ &\quad  + \theta \frac{1}{{\left({\sigma }_{T}^{2}+1\right)}^{2}}4{C}_{z}\left({\sigma }_{T}^{2}{\overline{Z} }^{4}{C}_{z}-{\sigma }_{z}^{2}{\sigma }_{T}^{2}{\overline{Z} }^{2}{\lambda }_{30}+{\sigma }_{T}^{2}{\overline{Z} }^{2}{\lambda }_{12}\left({\sigma }_{T}^{2}+1\right)\right).\end{aligned} $$

The generalized ratio estimator is as,

5 $${t}_{gratio}=\left(\left(\frac{{s}_{z}^{2}-{\sigma }_{s}^{2}-{\sigma }_{T}^{2}*{\overline{z} }^{2}}{{\sigma }_{T}^{2}+1}\right)+\left({\sigma }_{x}^{2}-{s}_{x}^{2}\right)\right)\times {\left(\frac{{(\alpha \sigma }_{x}^{2}+\beta )}{\omega \left({\alpha s}_{x}^{2}+\beta \right)+(1-\omega ){(\alpha \sigma }_{x}^{2}+\beta )}\right)}^{g}.$$

The MSE of *t*_0_ is as,

6 $$ \begin{aligned}\text{min }MSE\left({t}_{gratio}\right)&=\theta \left(\frac{{\sigma }_{z}^{4}\left({\mu }_{400}-1\right)+4{\sigma }_{T}^{2}{\overline{Z} }^{2}{C}_{z}^{2}-4{\sigma }_{z}^{2}{\sigma }_{T}^{2}{\overline{Z} }^{2}{\mu }_{300}{C}_{z}}{{({\sigma }_{T}^{2}+1)}^{2}}\right)\\ &\quad-\theta \left(\frac{1}{\left({\mu }_{040}-1\right)}\right){\left({\sigma }_{z}^{2}\left({\mu }_{220}-1\right)-2{\sigma }_{T}^{2}{\overline{Z} }^{2}{\mu }_{120}{C}_{z}\right)}^{2},\end{aligned} $$

where $${\mu }_{rsa}=\frac{{\mu }_{rsa}}{{\eta }_{200}^\frac{r}{2}{\eta }_{020}^\frac{s}{2}{\eta }_{020}^\frac{a}{2}}$$, $${\eta }_{rsa}=\frac{1}{N-1}\sum_{i=1}^{N}{({Y}_{i }- \overline{Y })}^{r}{({X}_{1i }- \overline{X })}^{s}{({X}_{2i }- \overline{X })}^{a}$$, $$\theta =\frac{1}{n}$$.

## The proposed estimator and its class of estimators

### The proposed estimator

In this section, a generalized exponential estimator is presented following Koyuncu et al.^11^. The form of the proposed estimator is given by,

7 $${t}_{1D}=\left[{k}_{1}\left(\frac{{s}_{z}^{2}-{\sigma }_{s}^{2}-{\sigma }_{T}^{2}{\overline{z} }^{2}}{{\sigma }_{T}^{2}+1}\right)+{k}_{2}\left({\sigma }_{x1}^{2}-{s}_{x1}^{2}\right)+{k}_{3}\left({\sigma }_{x2}^{2}-{s}_{x2}^{2}\right)\right]\left[{\left\{{\text{exp}}\left(\frac{{\sigma }_{x1}^{2}-{s}_{x1}^{2}}{{\sigma }_{x1}^{2}+{s}_{x1}^{2}}\right)\right\}}^{{\lambda }_{1}}{\left(\frac{{\sigma }_{x2}^{2}}{{s}_{x2}^{2}}\right)}^{{\lambda }_{2}}\right],$$

where ${k}_{1}$, ${k}_{2}$, ${k}_{3}$, are the three optimizing and unrestricted constants which need to be estimated such that the MSE of the estimator is minimum, and ${\lambda }_{1}$ and ${\lambda }_{2}$ are the generalization constants which need to be placed with some suitable values, known parameters, or function of known parameters to get different efficient and or existing estimators. A few examples are shown in Table 1 by setting different values to the constants.

**Table 1:** The class of estimators for different choices of constant’s values.

Class of estimators	${k}_{1}$	${k}_{2}$	${k}_{3}$	${\lambda }_{1}$	${\lambda }_{2}$
${t}_{1D(1)}=\left[{k}_{1}\left(\frac{{s}_{z}^{2}-{\sigma }_{s}^{2}-{\sigma }_{T}^{2}{\overline{z} }^{2}}{{\sigma }_{T}^{2}+1}\right)+{k}_{2}\left({\sigma }_{x1}^{2}-{s}_{x1}^{2}\right)+{k}_{3}\left({\sigma }_{x2}^{2}-{s}_{x2}^{2}\right)\right]{\text{exp}}\left(\frac{{\sigma }_{x1}^{2}-{s}_{x1}^{2}}{{\sigma }_{x1}^{2}+{s}_{x1}^{2}}\right)$	${k}_{1}$	${k}_{2}$	0	1	0
${t}_{1D(2)}=\left[{k}_{1}\left(\frac{{s}_{z}^{2}-{\sigma }_{s}^{2}-{\sigma }_{T}^{2}{\overline{z} }^{2}}{{\sigma }_{T}^{2}+1}\right)+{k}_{2}\left({\sigma }_{x1}^{2}-{s}_{x1}^{2}\right)+{k}_{3}\left({\sigma }_{x2}^{2}-{s}_{x2}^{2}\right)\right]\left(\frac{{\sigma }_{x2}^{2}}{{s}_{x2}^{2}}\right)$	${k}_{1}$	${k}_{2}$	0	0	1
${t}_{1D(3)}=\left(\frac{{s}_{z}^{2}-{\sigma }_{s}^{2}-{\sigma }_{T}^{2}{\overline{z} }^{2}}{{\sigma }_{T}^{2}+1}\right)\left(\frac{{\sigma }_{x2}^{2}}{{s}_{x2}^{2}}\right)$ Gupta et al.^28^	1	0	0	0	1
${t}_{1D(4)}=\left(\frac{{s}_{z}^{2}-{\sigma }_{s}^{2}-{\sigma }_{T}^{2}{\overline{z} }^{2}}{{\sigma }_{T}^{2}+1}\right)+{k}_{2}\left({\sigma }_{x1}^{2}-{s}_{x1}^{2}\right)+{k}_{3}\left({\sigma }_{x2}^{2}-{s}_{x2}^{2}\right)$	1	${k}_{2}$	*k*_*3*_	0	0
${t}_{1D(5)}=\left(\left(\frac{{s}_{z}^{2}-{\sigma }_{s}^{2}-{\sigma }_{T}^{2}{\overline{z} }^{2}}{{\sigma }_{T}^{2}+1}\right){+k}_{2}\left({\sigma }_{x1}^{2}-{s}_{x1}^{2}\right)\right){\text{exp}}\left(\frac{{\sigma }_{x1}^{2}-{s}_{x1}^{2}}{{\sigma }_{x1}^{2}+{s}_{x1}^{2}}\right)$	1	${k}_{2}$	0	1	0
${t}_{1D(6)}=\left[\left(\frac{{s}_{z}^{2}-{\sigma }_{s}^{2}-{\sigma }_{T}^{2}{\overline{z} }^{2}}{{\sigma }_{T}^{2}+1}\right)+{k}_{2}\left({\sigma }_{x1}^{2}-{s}_{x1}^{2}\right)\right]\left(\frac{{\sigma }_{x2}^{2}}{{s}_{x2}^{2}}\right)$	1	${k}_{2}$	0	0	1
${t}_{1D(7)}=\left(\frac{{s}_{z}^{2}-{\sigma }_{s}^{2}-{\sigma }_{T}^{2}{\overline{z} }^{2}}{{\sigma }_{T}^{2}+1}\right){\left\{{\text{exp}}\left(\frac{{\sigma }_{x1}^{2}-{s}_{x1}^{2}}{{\sigma }_{x1}^{2}+{s}_{x1}^{2}}\right)\right\}}^{{\lambda }_{1}}$	1	0	0	${\lambda }_{1}$	0
${t}_{1D(8)}=\left(\frac{{s}_{z}^{2}-{\sigma }_{s}^{2}-{\sigma }_{T}^{2}{\overline{z} }^{2}}{{\sigma }_{T}^{2}+1}\right){\left(\frac{{\sigma }_{x2}^{2}}{{s}_{x2}^{2}}\right)}^{{\lambda }_{2}}$	1	0	0	0	${\lambda }_{2}$

To obtain the Bias, and the MSE, we define the following error terms, rewriting Eq. (7), we have

8 $$ \begin{aligned}t_{1D} & = \left[ {k_{1} \left( {\frac{{\sigma_{z}^{2} \left( {1 + \delta_{z} } \right) - \sigma_{S}^{2} - \sigma_{T}^{2} \overline{Z}^{2} \left( {1 + e_{z} } \right)^{2} }}{{\sigma_{T}^{2} + 1}}} \right) + k_{2} \left( {\sigma_{x1}^{2} - \sigma_{x1}^{2} \left( {1 + \delta_{x1} } \right)} \right) + k_{3} \left( {\sigma_{x2}^{2} - \sigma_{x2}^{2} \left( {1 + \delta_{x2} } \right)} \right)} \right]\\ &\quad\left[ {\left\{ {\exp \left( {\frac{{\sigma_{x1}^{2} - \sigma_{x1}^{2} \left( {1 + \delta_{x1} } \right)}}{{\sigma_{x1}^{2} + \sigma_{x1}^{2} \left( {1 + \delta_{x1} } \right)}}} \right)} \right\}^{{\lambda_{1} }} \left( {\frac{{\sigma_{x2}^{2} }}{{\sigma_{x2}^{2} \left( {1 + \delta_{x2} } \right)}}} \right)^{{\lambda_{2} }} } \right].\end{aligned} $$

9 $$ \begin{aligned}Bias\left({t}_{1D}\right)&={\sigma }_{y}^{2}\left({k}_{1}-1\right)-{{k}_{1}\sigma }_{y}^{2}\theta \frac{1}{2}\left[{\lambda }_{1}\left\{{\lambda }_{2}\left({\mu }_{022}-1\right)-\frac{1}{4}{\lambda }_{1}\left({\mu }_{040}-1\right)\right\}-{\lambda }_{2}\frac{1}{2}\left({\lambda }_{2}-1\right)\left({\mu }_{004}-1\right)\right]\\ &\quad+{{k}_{1}\sigma }_{z}^{2}\theta \frac{1}{2{(\sigma }_{T}^{2}+1)}\left[{\lambda }_{1}\left({\mu }_{220}-1\right)-2{\lambda }_{2}\left({\mu }_{202}-1\right)\right]-\frac{{{\overline{Z} }^{2}\sigma }_{T}^{2}}{{(\sigma }_{T}^{2}+1)}{k}_{1}\theta \left[{C}_{z}+{\lambda }_{1}{\mu }_{120}-2{\mu }_{120}{\lambda }_{2}\right]{C}_{z}\\ &\quad-{k}_{2}{\sigma }_{x1}^{2}\theta \left[\frac{{\lambda }_{1}}{4}(\left({\mu }_{040}-1\right)-{\lambda }_{2}\left({\mu }_{022}-1\right)\right]-{k}_{3}{\sigma }_{x2}^{2}\theta \left[\frac{{\lambda }_{1}}{2}(\left({\mu }_{022}-1\right)-{\lambda }_{2}\left({\mu }_{004}-1\right)\right].\end{aligned} $$

The mean square error of the generalized estimator t_*1D*_ is given by

10 $$ \begin{aligned}MSE\left({t}_{1D}\right)&={\sigma }_{y}^{4}{\left({k}_{1}-1\right)}^{2}+{k}_{1}^{2}\theta {A}_{1}+{k}_{2}^{2}{\sigma }_{x1}^{4}\theta \left({\mu }_{040}-1\right)+{k}_{3}^{2}{\sigma }_{x2}^{4}\theta \left({\mu }_{004}-1\right)\\ &\quad+2{k}_{1}{k}_{2}{\sigma }_{x1}^{2}\theta {A}_{2}-2{k}_{1}{k}_{3}{\sigma }_{x2}^{2}\theta {A}_{3}+2{k}_{21}{k}_{3}{\sigma }_{x1}^{2}{\sigma }_{x2}^{2}\theta \left({\mu }_{022}-1\right).\end{aligned} $$

Differentiate Eq. (10) with respect to *k*_1_, *k*_2_, and* k*_3_, and after the simplification optimum values of the constants are given by,

$${k}_{11}=\frac{{\sigma }_{y}^{4}}{{A}_{7}}, {k}_{21}=\frac{-{k}_{1}{A}_{4}}{{\sigma }_{x1}^{2}{A}_{6}},\,\mathrm{ and }\,{k}_{31}=\frac{{k}_{1}{A}_{5}}{{\sigma }_{x2}^{2}{A}_{6}},$$

and utilizing the optimum values of the constants into Eq. (10), the simplified form of the minim MSE of the estimator is given by

11 $$\mathrm{min}\,MSE\left({t}_{1D}\right)={\sigma }_{y}^{6}\left(1-\frac{1}{{A}_{7}}\right),$$

where $${A}_{1}={\sigma }_{y}^{4}\left[\frac{{\lambda }_{1}^{2}}{4}\left({\mu }_{040}-1\right)+{\lambda }_{2}^{2}\left({\mu }_{004}-1\right)-{\lambda }_{1}{\lambda }_{2}\left({\mu }_{022}-1\right)\right]+\frac{{\sigma }_{z}^{4}\left({\mu }_{400}-1\right)}{{{(\sigma }_{T}^{2}+1)}^{2}}+4\frac{{\overline{Z} }^{4}{\sigma }_{T}^{4}{C}_{z}^{2}}{{{(\sigma }_{T}^{2}+1)}^{2}}+\frac{{{\overline{Z} }^{2}\sigma }_{T}^{2}{\sigma }_{z}^{2}{\mu }_{300}{C}_{z}}{{(\sigma }_{T}^{2}+1)}-2\frac{{\sigma }_{y}^{2}}{{\sigma }_{T}^{2}+1}\left[{\sigma }_{z}^{2}\left\{\frac{{\lambda }_{1}}{2}(\left({\mu }_{220}-1\right)-{\lambda }_{2}\left({\mu }_{202}-1\right)\right\}+2{{\overline{Z} }^{2}\sigma }_{T}^{2}\left\{\frac{{\lambda }_{1}}{2}{\mu }_{120}-{{\lambda }_{2}\mu }_{102}\right\}{C}_{z}\right]$$, $${A}_{2}={\sigma }_{y}^{2}\left[\frac{{\lambda }_{1}}{2}\left({\mu }_{040}-1\right)-{\lambda }_{2}\left({\mu }_{022}-1\right)\right]-\frac{{\sigma }_{z}^{2}\left({\mu }_{220}-1\right)}{{(\sigma }_{T}^{2}+1)}+2\frac{{{\overline{Z} }^{2}\sigma }_{T}^{2}{\mu }_{120}}{{(\sigma }_{T}^{2}+1)}{C}_{z}$$, $${A}_{3}={\sigma }_{y}^{2}\left[\frac{{\lambda }_{1}}{2}\left({\mu }_{022}-1\right)-{\lambda }_{2}\left({\mu }_{004}-1\right)\right]-\frac{{\sigma }_{z}^{2}\left({\mu }_{202}-1\right)}{{(\sigma }_{T}^{2}+1)}+2\frac{{{\overline{Z} }^{2}\sigma }_{T}^{2}{\mu }_{120}}{{(\sigma }_{T}^{2}+1)}{C}_{z}$$, $${A}_{4}={A}_{2}\left({\mu }_{004}-1\right)+{A}_{3}\left({\mu }_{022}-1\right)$$, $${A}_{5}={A}_{2}\left({\mu }_{022}-1\right)+{A}_{3}\left({\mu }_{040}-1\right)$$, $${A}_{6}=\left({\mu }_{040}-1\right)\left({\mu }_{004}-1\right)-{\left({\mu }_{022}-1\right)}^{2}$$, and $${A}_{7}={\sigma }_{y}^{4}+\theta {A}_{1}+\theta \left({\mu }_{040}-1\right){\left(\frac{{A}_{4}}{{A}_{6}}\right)}^{2}+\theta \left({\mu }_{004}-1\right){\left(\frac{{A}_{5}}{{A}_{6}}\right)}^{2}+2\theta \frac{{A}_{2}{A}_{4}}{{A}_{6}}-2\theta \frac{{A}_{3}{A}_{5}}{{A}_{6}}+2\theta \left({\mu }_{022}-1\right)\frac{{A}_{4}{A}_{5}}{{A}_{6}^{2}}$$.

### Mathematical comparison of the proposed class of estimators with ${t}_{0}$

i. $MSE\left({t}_{0}\right)-MSE\left({t}_{1D(1)}\right)=\frac{{B}_{1}}{{B}_{2}}>1$ is true iff ${B}_{2}$ > ${B}_{1}$, where ${B}_{1}=\left(\frac{1}{{({\sigma }_{T}^{2}+1)}^{2}}\right)\left({\sigma }_{z}^{4}({\mu }_{400}-1)+4{\sigma }_{T}^{2}{\overline{Z} }^{2}{C}_{z}^{2}-4{\sigma }_{z}^{2}{\sigma }_{T}^{2}{\overline{Z} }^{2}{\mu }_{300}{C}_{z}\right)$, ${B}_{2}=\frac{{\sigma }_{y}^{4}}{\theta }{\left({k}_{1}-1\right)}^{2}+{k}_{1}^{2}{B}_{21}+{k}_{2}^{2}{\sigma }_{x1}^{4}\left({\mu }_{040}-1\right)+2{k}_{1}{k}_{2}{\sigma }_{x1}^{2}{B}_{22}$, ${B}_{21}=\frac{{\sigma }_{y}^{4}}{4}\left({\mu }_{040}-1\right)+\frac{{\sigma }_{z}^{4}\left({\mu }_{400}-1\right)}{{{(\sigma }_{T}^{2}+1)}^{2}}+4\frac{{\overline{Z} }^{4}{\sigma }_{T}^{4}{C}_{z}^{2}}{{{(\sigma }_{T}^{2}+1)}^{2}}+\frac{{{\overline{Z} }^{2}\sigma }_{T}^{2}{\sigma }_{z}^{2}{\mu }_{300}{C}_{z}}{{(\sigma }_{T}^{2}+1)}-2\frac{{\sigma }_{y}^{2}}{{\sigma }_{T}^{2}+1}\left[\frac{{\sigma }_{z}^{2}}{2}(\left({\mu }_{220}-1\right)+{{\overline{Z} }^{2}\sigma }_{T}^{2}{{\mu }_{120}C}_{z}\right]$, ${B}_{22}=\frac{{\sigma }_{y}^{2}}{2}\left({\mu }_{040}-1\right)-\frac{{\sigma }_{z}^{2}\left({\mu }_{220}-1\right)}{{(\sigma }_{T}^{2}+1)}+2\frac{{{\overline{Z} }^{2}\sigma }_{T}^{2}{\mu }_{120}}{{(\sigma }_{T}^{2}+1)}{C}_{z}$.
ii. $MSE\left({t}_{0}\right)-MSE\left({t}_{1D(2)}\right)=\frac{{B}_{1}}{{B}_{3}}>1$ is true iff ${B}_{3}$ > ${B}_{1}$, where ${B}_{3}=\frac{{\sigma }_{y}^{4}}{\theta }{\left({k}_{1}-1\right)}^{2}+{k}_{1}^{2}{B}_{31}+{k}_{2}^{2}{\sigma }_{x1}^{4}\left({\mu }_{040}-1\right)+2{k}_{1}{k}_{2}{\sigma }_{x1}^{2}{B}_{32}$, ${B}_{31}={\sigma }_{y}^{4}{\lambda }_{2}^{2}\left({\mu }_{004}-1\right)+\frac{{\sigma }_{z}^{4}\left({\mu }_{400}-1\right)}{{{(\sigma }_{T}^{2}+1)}^{2}}+4\frac{{\overline{Z} }^{4}{\sigma }_{T}^{4}{C}_{z}^{2}}{{{(\sigma }_{T}^{2}+1)}^{2}}+\frac{{{\overline{Z} }^{2}\sigma }_{T}^{2}{\sigma }_{z}^{2}{\mu }_{300}{C}_{z}}{{(\sigma }_{T}^{2}+1)}+2\frac{{\sigma }_{y}^{2}}{{\sigma }_{T}^{2}+1}\left[{\sigma }_{z}^{2}{\lambda }_{2}\left({\mu }_{202}-1\right)+2{{\overline{Z} }^{2}\sigma }_{T}^{2}{{\lambda }_{2}\mu }_{102}{C}_{z}\right]$, ${B}_{32}=2\frac{{{\overline{Z} }^{2}\sigma }_{T}^{2}{\mu }_{120}}{{(\sigma }_{T}^{2}+1)}{C}_{z}-{\sigma }_{y}^{2}{\lambda }_{2}\left({\mu }_{022}-1\right)-\frac{{\sigma }_{z}^{2}\left({\mu }_{220}-1\right)}{{(\sigma }_{T}^{2}+1)}.$
iii. $MSE\left({t}_{0}\right)-\left[MSE\left({t}_{1D(3)}\right)=MSE\left({t}_{ratio}\right)\right]=\frac{{B}_{1}}{{B}_{4}}>1$ is true iff ${B}_{4}$>${B}_{1}$, where ${B}_{4}={\sigma }_{y}^{4}{\lambda }_{2}^{2}\left({\mu }_{004}-1\right)+\frac{{\sigma }_{z}^{4}\left({\mu }_{400}-1\right)}{{{(\sigma }_{T}^{2}+1)}^{2}}+4\frac{{\overline{Z} }^{4}{\sigma }_{T}^{4}{C}_{z}^{2}}{{{(\sigma }_{T}^{2}+1)}^{2}}+\frac{{{\overline{Z} }^{2}\sigma }_{T}^{2}{\sigma }_{z}^{2}{\mu }_{300}{C}_{z}}{{(\sigma }_{T}^{2}+1)}+2\frac{{\sigma }_{y}^{2}}{{\sigma }_{T}^{2}+1}\left[{\sigma }_{z}^{2}{\lambda }_{2}\left({\mu }_{202}-1\right)+2{{\overline{Z} }^{2}\sigma }_{T}^{2}{{\lambda }_{2}\mu }_{102}{C}_{z}\right]$.
iv. $MSE\left({t}_{0}\right)-MSE\left({t}_{1D(4)}\right)=\frac{{B}_{1}}{{B}_{5}}>1$ is true iff ${B}_{5}$ > ${B}_{1}$, where ${B}_{5}=\left[\frac{{\sigma }_{z}^{4}\left({\mu }_{400}-1\right)}{{{(\sigma }_{T}^{2}+1)}^{2}}+4\frac{{\overline{Z} }^{4}{\sigma }_{T}^{4}{C}_{z}^{2}}{{{(\sigma }_{T}^{2}+1)}^{2}}+\frac{{{\overline{Z} }^{2}\sigma }_{T}^{2}{\sigma }_{z}^{2}{\mu }_{300}{C}_{z}}{{(\sigma }_{T}^{2}+1)}\right]+{k}_{2}^{2}{\sigma }_{x1}^{4}\left({\mu }_{040}-1\right)+{k}_{3}^{2}{\sigma }_{x2}^{4}\left({\mu }_{004}-1\right)+2{k}_{2}{\sigma }_{x1}^{2}\left[2\frac{{{\overline{Z} }^{2}\sigma }_{T}^{2}{\mu }_{120}}{{(\sigma }_{T}^{2}+1)}{C}_{z}-\frac{{\sigma }_{z}^{2}\left({\mu }_{220}-1\right)}{{(\sigma }_{T}^{2}+1)}\right]-2{k}_{1}{k}_{3}{\sigma }_{x2}^{2}\left[2\frac{{{\overline{Z} }^{2}\sigma }_{T}^{2}{\mu }_{120}}{{(\sigma }_{T}^{2}+1)}{C}_{z}-\frac{{\sigma }_{z}^{2}\left({\mu }_{202}-1\right)}{{(\sigma }_{T}^{2}+1)}\right]+2{k}_{3}{\sigma }_{x1}^{2}{\sigma }_{x2}^{2}\left({\mu }_{022}-1\right)$.
v. $MSE\left({t}_{0}\right)-MSE\left({t}_{1D(5)}\right)=\frac{{B}_{1}}{{B}_{6}}>1$ is true iff ${B}_{6}$ > ${B}_{1}$, where ${B}_{6}={B}_{21}+{k}_{2}^{2}{\sigma }_{x1}^{4}\left({\mu }_{040}-1\right)+2{k}_{2}{\sigma }_{x1}^{2}{B}_{22}$.
vi. $MSE\left({t}_{0}\right)-MSE\left({t}_{1D(7)}\right)=\frac{{B}_{1}}{{B}_{7}}>1$ is true iff ${B}_{7}$ > ${B}_{1}$, where ${B}_{7}={B}_{31}+{k}_{2}^{2}{\sigma }_{x1}^{4}\left({\mu }_{040}-1\right)+2{k}_{2}{\sigma }_{x1}^{2}{B}_{32}$.
vii. $MSE\left({t}_{0}\right)-MSE\left({t}_{1D(8)}\right)=\frac{{B}_{1}}{{B}_{8}}>1$ is true iff ${B}_{8}$ > ${B}_{1}$, where ${B}_{8}={\sigma }_{y}^{4}\left[\frac{{\lambda }_{1}^{2}}{4}\left({\mu }_{040}-1\right)+\left({\mu }_{004}-1\right)-{\lambda }_{1}\left({\mu }_{022}-1\right)\right]+\frac{{\sigma }_{z}^{4}\left({\mu }_{400}-1\right)}{{{(\sigma }_{T}^{2}+1)}^{2}}+4\frac{{\overline{Z} }^{4}{\sigma }_{T}^{4}{C}_{z}^{2}}{{{(\sigma }_{T}^{2}+1)}^{2}}+\frac{{{\overline{Z} }^{2}\sigma }_{T}^{2}{\sigma }_{z}^{2}{\mu }_{300}{C}_{z}}{{(\sigma }_{T}^{2}+1)}-2\frac{{\sigma }_{y}^{2}}{{\sigma }_{T}^{2}+1}\left[{\sigma }_{z}^{2}\left\{\frac{{\lambda }_{1}}{2}(\left({\mu }_{220}-1\right)-\left({\mu }_{202}-1\right)\right\}+2{{\overline{Z} }^{2}\sigma }_{T}^{2}\left\{\frac{{\lambda }_{1}}{2}{\mu }_{120}-{\mu }_{102}\right\}{C}_{z}\right]$.
viii. $MSE\left({t}_{0}\right)-MSE\left({t}_{1D(9)}\right)=\frac{{B}_{1}}{{B}_{9}}>1$ is true iff ${B}_{9}$ > ${B}_{1}$, where ${B}_{9}={\sigma }_{y}^{4}{\lambda }_{2}^{2}\left({\mu }_{004}-1\right)+\frac{{\sigma }_{z}^{4}\left({\mu }_{400}-1\right)}{{{(\sigma }_{T}^{2}+1)}^{2}}+4\frac{{\overline{Z} }^{4}{\sigma }_{T}^{4}{C}_{z}^{2}}{{{(\sigma }_{T}^{2}+1)}^{2}}+\frac{{{\overline{Z} }^{2}\sigma }_{T}^{2}{\sigma }_{z}^{2}{\mu }_{300}{C}_{z}}{{(\sigma }_{T}^{2}+1)}+2\frac{{\sigma }_{y}^{2}}{{\sigma }_{T}^{2}+1}\left[{\sigma }_{z}^{2}{\lambda }_{2}\left({\mu }_{202}-1\right)+2{{\overline{Z} }^{2}\sigma }_{T}^{2}{{\lambda }_{2}\mu }_{102}{C}_{z}\right]$.

The above (i)–(vii) expressions the conditions under which the proposed class of estimators performs better as compared to the estimators ${t}_{0}$.

## The proposed RRT model and estimator-II

### The proposed RRT model

Our scrambled randomized response model provides a combination of multiplicative, additive, and subtractive models. Since *Y* is the sensitive variable of interest and hence subject to social desirability bias. *S* and *R* are the two independent scrambling variables and are mutually uncorrelated with *Y*. We assume

12 $${Z}_{NP}=g\left(Y+aS\right)+\left(1-g\right)R\left(Y+aS\right),$$

13 $${\sigma }_{{Z}_{NP}}^{2}={g}^{2}\left({\sigma }_{Y}^{2}+{a}^{2}{\sigma }_{S}^{2}\right)+(1-{g)}^{2}{\sigma }_{R}^{2}\left({\sigma }_{Y}^{2}+{a}^{2}{\sigma }_{S}^{2}\right),$$

14 $$\begin{aligned} \sigma_{{Z_{NP} }}^{2} = & g^{2} \left( {\sigma_{Y}^{2} + a^{2} \sigma_{S}^{2} } \right) + (1 - g)^{2} \sigma_{RY}^{2} + a^{2} (1 - g)^{2} \sigma_{RS}^{2} \\ = & g^{2} \left( {\sigma_{Y}^{2} + a^{2} \sigma_{S}^{2} } \right) + (1 - g)^{2} \left( {\left( {\sigma_{R}^{2} \sigma_{Y}^{2} + \sigma_{R}^{2} } \right)\left( {E\left[ Y \right]} \right)^{2} + \sigma_{Y}^{2} \left( {E\left[ R \right]} \right)^{2} } \right) \\ & + \;a^{2} (1 - g)^{2} \left( {\sigma_{R}^{2} \sigma_{S}^{2} + \sigma_{R}^{2} \left( {E\left[ S \right]} \right)^{2} + \sigma_{S}^{2} \left( {E\left[ R \right]} \right)^{2} } \right) \\ = & \sigma_{Y}^{2} \left[ {g^{2} + (1 - g)^{2} \left( {\sigma_{R}^{2} + 1} \right)} \right] + a^{2} \sigma_{S}^{2} \left[ {g^{2} + (1 - g)^{2} \left( {\sigma_{R}^{2} + 1} \right)} \right] + (1 - g)^{2} \sigma_{R}^{2} \mu_{Y}^{2} . \\ \end{aligned}$$

Rewriting, we get

15 $${\sigma }_{Y}^{2}=\frac{{\sigma }_{{Z}_{NP}}^{2}-(1-{g)}^{2}{\sigma }_{R}^{2}{\overline{Z} }^{2}}{\left[{g}^{2}+(1-{g)}^{2}\left({\sigma }_{R}^{2}+1\right)\right]}-{a}^{2}{\sigma }_{S}^{2}.$$

(i) Estimating ${\sigma }_{ZNP }^{2}$ by its unbiased estimator ${s}_{Z }^{2}$,
  16 $${t}_{NP1}=\frac{{s}_{Z}^{2}-(1-{g)}^{2}{\sigma }_{R}^{2}*{\overline{z} }^{2}}{\left[{g}^{2}+(1-{g)}^{2}\left({\sigma }_{R}^{2}+1\right)\right]}-{a}^{2}{\sigma }_{S}^{2}.$$
  Rewrite (16) we get
  17 $${t}_{NP1}=\frac{{\sigma }_{Z}^{2}(1-{\delta }_{Z})-(1-{g)}^{2}{\sigma }_{R}^{2}{\overline{Z} }^{2}{\left(1+{e}_{0}\right)}^{2}}{\left[{g}^{2}+(1-{g)}^{2}\left({\sigma }_{R}^{2}+1\right)\right]}-{a}^{2}{\sigma }_{S}^{2},$$
  18 $${t}_{NP1}=\frac{{\sigma }_{Z}^{2}-(1-{g)}^{2}{\sigma }_{R}^{2}{\overline{Z} }^{2}}{\left[{g}^{2}+(1-{g)}^{2}\left({\sigma }_{R}^{2}+1\right)\right]}-{a}^{2}{\sigma }_{S}^{2}+\frac{{\sigma }_{Z}^{2}{\delta }_{Z}-(1-{g)}^{2}{\sigma }_{R}^{2}{\overline{Z} }^{2}({e}_{0}^{2}+2{e}_{0})}{\left[{g}^{2}+(1-{g)}^{2}\left({\sigma }_{R}^{2}+1\right)\right]},$$
  19 $${t}_{NP1}-{\sigma }_{Y}^{2}=\frac{{\sigma }_{Z}^{2}{\delta }_{Z}-(1-{g)}^{2}{\sigma }_{R}^{2}{\overline{Z} }^{2}({e}_{0}^{2}+2{e}_{0})}{\left[{g}^{2}+(1-{g)}^{2}\left({\sigma }_{R}^{2}+1\right)\right]}.$$
  By pertaining expectations together on (19), the bias we obtain is as
  20 $$Bias\, \left({t}_{NP1}\right)=-\theta \frac{(1-{g)}^{2}{\sigma }_{R}^{2}{\overline{Z} }^{2}}{\left[{g}^{2}+(1-{g)}^{2}\left({\sigma }_{R}^{2}+1\right)\right]}{C}_{Z}^{2},$$
  21 $$MSE \left({t}_{NP1}\right)=\theta \frac{1}{{\left[{g}^{2}+(1-{g)}^{2}\left({\sigma }_{R}^{2}+1\right)\right]}^{2}}\left[{\sigma }_{Z}^{4}\left({\lambda }_{400}-1\right)+4(1-{g)}^{4}{\sigma }_{R}^{4}{\overline{Z} }^{2}{C}_{Z}^{2}-4(1-{g)}^{2}{\sigma }_{R}^{2}{\overline{Z} }^{2}{\lambda }_{300}{C}_{Z}\right].$$
(ii) Ratio estimator under the proposed randomized model:
  22 $${t}_{NP2}=\left[\frac{{s}_{Z}^{2}-(1-{g)}^{2}{\sigma }_{R}^{2}\times {\overline{z} }^{2}}{\left[{g}^{2}+(1-{g)}^{2}\left({\sigma }_{R}^{2}+1\right)\right]}-{a}^{2}{\sigma }_{S}^{2}\right]\left[\frac{{\sigma }_{X1}^{2}}{{s}_{X1}^{2}}\right].$$
  Rewrite (22) we get
  23 $${t}_{NP2}=\left[\frac{{\sigma }_{Z}^{2}(1-{\delta }_{Z})-(1-{g)}^{2}{\sigma }_{R}^{2}{\overline{Z} }^{2}{\left(1+{e}_{0}\right)}^{2}}{\left[{g}^{2}+(1-{g)}^{2}\left({\sigma }_{R}^{2}+1\right)\right]}-{a}^{2}{\sigma }_{S}^{2}\right]\left[\frac{{\sigma }_{X1}^{2}}{{\sigma }_{X1}^{2}(1-{\delta }_{X1})}\right],$$
  24 $${t}_{NP2}=\left[{\sigma }_{Y+}^{2}\frac{{\sigma }_{Z}^{2}{\delta }_{Z}-(1-{g)}^{2}{\sigma }_{R}^{2}{\overline{Z} }^{2}({e}_{0}^{2}+2{e}_{0})}{\left[{g}^{2}+(1-{g)}^{2}\left({\sigma }_{R}^{2}+1\right)\right]}\right]{(1-{\delta }_{X1})}^{-1}.$$
  By pertaining expectations together on (24), the Bias and mean square error we obtain are as
  25 $$Bias\, \left({t}_{NP2}\right)=\frac{\theta }{G}\left[\left({\lambda }_{040}-1\right)G-\left({\sigma }_{Z}^{4}{\lambda }_{120}{C}_{Z}+(1-{g)}^{2}{\sigma }_{R}^{2}{\overline{Z} }^{2}\left({C}_{Z}-2{\lambda }_{120}\right)\right){C}_{Z}\right],$$
  26 $$MSE \left({t}_{NP2}\right)=\frac{\theta }{G}\left[\begin{array}{c}\frac{1}{G}\left\{{\sigma }_{Z}^{4}\left({\lambda }_{400}-1\right)G-(1-{g)}^{2}{\sigma }_{R}^{2}{\overline{Z} }^{2}{\left(2{\lambda }_{300}-(1-{g)}^{2}{\sigma }_{R}^{2}{\overline{Z} }^{2}\right)C}_{Z}\right\}\\ -{\sigma }_{Y}^{2}\left\{{\sigma }_{Z}^{2}\left({\lambda }_{220}-1\right)+(1-{g)}^{2}{\sigma }_{R}^{2}{\overline{Z} }^{2}{\lambda }_{120}{C}_{Z}-{\sigma }_{Z}^{2}\left({\lambda }_{040}-1\right)\right\}\end{array}\right],$$
  where
  $$G=\left[{g}^{2}+(1-{g)}^{2}\left({\sigma }_{R}^{2}+1\right)\right].$$

### The proposed estimator under the proposed RRT model

The exponential estimator expressed in (7) can be generalized in the situation of two auxiliary non-sensitive variables as,

27 $${t}_{1N}=\left[{w}_{1}\left(\frac{{s}_{{Z}_{NP}}^{2}-(1-{g)}^{2}{\sigma }_{R}^{2}{\overline{z} }^{2}}{\left[{g}^{2}+(1-{g)}^{2}\left({\sigma }_{R}^{2}+1\right)\right]}-{a}^{2}{\sigma }_{S}^{2} \right)+{w}_{2}\left({\sigma }_{x1}^{2}-{s}_{x1}^{2}\right)+{w}_{3}\left({\sigma }_{x2}^{2}-{s}_{x2}^{2}\right)\right]\left[{\left\{{\text{exp}}\left(\frac{{\sigma }_{x1}^{2}-{s}_{x1}^{2}}{{\sigma }_{x1}^{2}+{s}_{x1}^{2}}\right)\right\}}^{{v}_{1}}{\left(\frac{{\sigma }_{x2}^{2}}{{s}_{x2}^{2}}\right)}^{{v}_{2}}\right].$$

Rewrite (27) we get,

28 $$ \begin{aligned}{t}_{1N}&=\left[{w}_{1}\left\{\frac{{\sigma }_{Z}^{2}(1-{\delta }_{Z})-(1-{g)}^{2}{\sigma }_{R}^{2}{\overline{Z} }^{2}{\left(1+{e}_{0}\right)}^{2}}{\left[{g}^{2}+(1-{g)}^{2}\left({\sigma }_{R}^{2}+1\right)\right]}-{a}^{2}{\sigma }_{S}^{2} \right\}\right.\\&\quad\left. +{w}_{2}\left({\sigma }_{x1}^{2}-{\sigma }_{x1}^{2}\left(1-{\delta }_{x1}\right)\right)+{w}_{3}\left({\sigma }_{x2}^{2}-{\sigma }_{x2}^{2}(1-{\delta }_{x2})\right)\vphantom{\left\{\frac{{\sigma }_{Z}^{2}(1-{\delta }_{Z})-(1-{g)}^{2}{\sigma }_{R}^{2}{\overline{Z} }^{2}{\left(1+{e}_{0}\right)}^{2}}{\left[{g}^{2}+(1-{g)}^{2}\left({\sigma }_{R}^{2}+1\right)\right]}-{a}^{2}{\sigma }_{S}^{2} \right\}}\right]\\ &\quad\left[{\left\{{\text{exp}}\left(\frac{{\sigma }_{x1}^{2}-{\sigma }_{x1}^{2}(1-{\delta }_{x1})}{{\sigma }_{x1}^{2}+{\sigma }_{x1}^{2}(1-{\delta }_{x1})}\right)\right\}}^{{v}_{1}}{\left(\frac{{\sigma }_{x2}^{2}}{{\sigma }_{x2}^{2}(1-{\delta }_{x2})}\right)}^{{v}_{2}}\right].\end{aligned} $$

The expression of the bias and MSE of ${t}_{1N}$ to the first order of approximation is given by,

29 $$ \begin{aligned}{Bias(t}_{1N})&={\sigma }_{y}^{2}\left({w}_{1}-1\right)-{w}_{1}\theta \left[(1-{g)}^{2}{\overline{Z} }^{2}{\sigma }_{R}^{2}{C}_{Z}\left(\frac{{C}_{z}}{{g}^{2}+(1-{g)}^{2}\left({\sigma }_{R}^{2}+1\right)}+{\mu }_{120}\right)\right. \\ & \quad \left. +{v}_{1}\frac{1}{2}\left({\sigma }_{z}^{2}\left({\mu }_{022}-1\right)+{v}_{1}\frac{1}{4}\left({\mu }_{040}-1\right)\right)-{v}_{2}{\sigma }_{Y}^{2}\left({\mu }_{004}-1\right)\right]\\ &\quad+\theta \frac{1}{2}\left({\mu }_{004}-1\right)\left[{w}_{2}{\sigma }_{x1}^{2}{v}_{1}+{v}_{2}\left({v}_{2}-1\right){\sigma }_{Y}^{2}\right]-{k}_{3}{\sigma }_{x2}^{2}\theta \frac{1}{2}\left({\mu }_{022}-1\right),\end{aligned} $$

30 $$ \begin{aligned}{MSE(t}_{1N})&={\sigma }_{y}^{4}{\left({w}_{1}-1\right)}^{2}+{w}_{1}^{2}\theta {B}_{1}+{k}_{2}^{2}{\sigma }_{x1}^{4}\theta \left({\mu }_{040}-1\right)\\ &\quad+\frac{1}{4}{k}_{3}^{2}{\sigma }_{x2}^{4}\theta \left({\mu }_{004}-1\right)+2{k}_{1}{k}_{2}{\sigma }_{x1}^{2}\theta {B}_{2}-2{k}_{1}{k}_{23}{\sigma }_{x2}^{2}\theta {B}_{3}\\ &\quad+{k}_{12}{k}_{23}{\sigma }_{x1}^{2}{\sigma }_{x2}^{2}\theta \left({\mu }_{022}-1\right).\end{aligned} $$

Differentiate with respect to ${w}_{1},{w}_{2}$ and ${w}_{3}$, the optimum values attained are as ${w}_{1}=\frac{{\sigma }_{y}^{4}}{{\sigma }_{y}^{4}+\theta {B}_{8}}$, ${w}_{2}=\frac{-{w}_{1}{B}_{4}}{{\sigma }_{x1}^{2}{B}_{5}}$ and ${w}_{3}=\frac{{w}_{1}{B}_{6}}{{\sigma }_{x2}^{2}{B}_{7}}.$

The MSE imputing this optimum value is given as,

31 $$\mathrm{min }MSE\left({t}_{1N}\right)=1-\frac{{\sigma }_{y}^{6}}{{\sigma }_{y}^{4}+\theta {B}_{8}},$$

where $${D}_{1}=\frac{1}{{\left[{g}^{2}+{\left(1-g\right)}^{2}\left({\sigma }_{R}^{2}+1\right)\right]}^{2}}\left[{\sigma }_{z}^{4}\left({\mu }_{400}-1\right)+{4\left(1-g\right)}^{4}{\sigma }_{z}^{4}{\overline{Z} }^{4}{C}_{z}^{2}-2{\left(1-g\right)}^{2}{\sigma }_{z}^{2}{\sigma }_{R}^{2}{\overline{Z} }^{2}{\mu }_{300}{C}_{z}\right]$$, $${D}_{2}=\frac{1}{\left[{g}^{2}+{\left(1-g\right)}^{2}\left({\sigma }_{R}^{2}+1\right)\right]}\left[{\sigma }_{z}^{2}\{{v}_{1}\left({\mu }_{220}-1\right)+{v}_{2}\left({\mu }_{202}-1\right)\}-{2\left(1-g\right)}^{2}{\sigma }_{R}^{2}{\overline{Z} }^{2}\{{v}_{1}{\mu }_{120}+{v}_{2}{\mu }_{102}\}{C}_{z}\right]$$, $${D}_{3}={v}_{1}^{2}\left({\mu }_{040}-1\right)+{v}_{2}^{2}\left({\mu }_{004}-1\right)-2{v}_{1}{v}_{2}\left({\mu }_{022}-1\right)$$, $${D}_{4}=\theta \left({D}_{1}+{\sigma }_{y}^{4}{D}_{2}-2{\sigma }_{y}^{2}{D}_{3}\right)$$, $${B}_{1}={D}_{1}+{\sigma }_{y}^{4}\left[\frac{{v}_{1}^{2}}{2}\left({\mu }_{040}-1\right)+{v}_{2}^{2}\left({\mu }_{004}-1\right)-2{v}_{1}{v}_{2}\left({\mu }_{022}-1\right)\right]-{\sigma }_{y}^{2}\left[{v}_{1}{D}_{2}-{v}_{2}{D}_{3}+2{v}_{1}{v}_{2}\left({\mu }_{022}-1\right)\right]$$, $${B}_{2}= {D}_{2}+\left[{\sigma }_{y}^{2}\left({\mu }_{040}-1\right)+2\left({\mu }_{022}-1\right)\right]{v}_{1}$$, $${B}_{3}= {D}_{3}+\left[{\sigma }_{y}^{2}\left({\mu }_{022}-1\right)+2\left({\mu }_{004}-1\right)\right]{v}_{2}$$*,*$${B}_{4}=\left[{B}_{2}\left({\mu }_{004}-1\right)-{B}_{3}\left({\mu }_{022}-1\right)\right],$$ $${B}_{5}={\sigma }_{X1}^{2}\left[\left({\mu }_{040}-1\right)\left({\mu }_{004}-1\right)-{\left({\mu }_{022}-1\right)}^{2}\right],$$ $${B}_{6}=\left[{B}_{2}\left({\mu }_{022}-1\right)-{B}_{3}\left({\mu }_{040}-1\right)\right],$$ $${B}_{7}={\sigma }_{X2}^{2}\left[\left({\mu }_{004}-1\right)\left({\mu }_{040}-1\right)-{\left({\mu }_{022}-1\right)}^{2}\right],$$ and,

$${B}_{8}={B}_{1}+\left({\mu }_{040}-1\right){\left[\frac{{B}_{4}}{{B}_{5}}\right]}^{2}+\left({\mu }_{004}-1\right){\left[\frac{{B}_{6}}{{B}_{7}}\right]}^{2}-2\left[\frac{{B}_{2}{B}_{4}}{{B}_{5}}-\frac{{B}_{3}{B}_{6}}{{B}_{7}}+\left({\mu }_{022}-1\right)\frac{{B}_{4}{B}_{6}}{{B}_{5}{B}_{7}}\right].$$

## Privacy levels

In the literature, many privacy protection measures are presented by different authors. For our study, privacy measure due to Yan et al.^30^ is used to compute privacy for Diana and Perri’s^6^ model, and the proposed randomized response model.

The privacy protection measure presented by Yan et al. is given by,

32 $$\Delta ={E\left({Z}_{i}-{Y}_{i}\right)}^{2}.$$

- Diana and Perri^6^ model is given by,
  33 $$Z=RY+S.$$
  The privacy protection level is given by
  34 $$\begin{aligned} \Delta_{D} = & E\left( {Z_{i} - Y_{i} } \right)^{2} \\ = & \sigma_{R}^{2} \left( {\mu_{Y}^{2} + \sigma_{y}^{2} } \right) + \sigma_{s}^{2} . \\ \end{aligned}$$
- The proposed Randomized response model’s privacy protection mlevel is as,
  35 $$\begin{aligned} \Delta_{PN} = & E\left( {Z_{i} - Y_{i} } \right)^{2} \\ = & \left( {\mu_{Y}^{2} + \sigma_{y}^{2} + a^{2} \sigma_{s}^{2} } \right)\left( {1 + \left( {1 - g} \right)^{2} \sigma_{R}^{2} } \right) - \left( {\mu_{Y}^{2} + \sigma_{y}^{2} } \right). \\ \end{aligned}$$
- Comparison of privacy protection levels for Diana and Perris’^6^ model and proposed model.
  Using (34) and (35), we have
  36 $${\Delta }_{PN}-{\Delta }_{D}=\left({\mu }_{Y}^{2}+{\sigma }_{y}^{2}\right){\sigma }_{R}^{2}g\left(g-2\right)-{\sigma }_{s}^{2}\left[1-{a}^{2}\left(1+{\left(1-g\right)}^{2}{\sigma }_{R}^{2}\right)\right]>0,$$
  $$\mathrm{iff }g\left(g-2\right)>\frac{{\sigma }_{s}^{2}\left[1-{a}^{2}\left(1+{\sigma }_{R}^{2}\right)\right]}{\left({\sigma }_{R}^{2}\left({\mu }_{Y}^{2}+{\sigma }_{y}^{2}\right)-{a}^{2}{\sigma }_{s}^{2}{\sigma }_{R}^{2}\right)}.$$

## An application of the proposed model

In this section, motivated by Saleem and Sanaullah^17^ a real-life application is presented to analyze the efficiency of the proposed RRT model compared to the existing models.

A survey is organized to collect real data for the problem of the estimation of the true variance of the Grade Point Average (GPA) of the students of the Department of Statistics, in Forman Christian College University Lahore, who have studied the Course: *Statistical Methods* in Spring 2023. Ninety students registered in three sections in this statistics course are considered as our population. In this application, the variable of interest Y is the CGPA of students, and the two auxiliary variables i.e., X_1_ is the weekly study hours, and X_2_ is the number of courses studied in recent semesters. For the scrambling variables, S is a normal random variable with a mean equal to zero and a standard deviation equal to 2, and R is a normal random variable with a mean equal to 1 and a standard deviation equal to 0.02. The following are some characteristics of the population:

$${\mathrm{N}=90, \mu }_{{X}_{1}}=27.61;{\mu }_{{X}_{2}}=19.88;{\sigma }_{{X}_{1}}=8.66;{\sigma }_{{X}_{2}}=18.83.$$

For model, $$Z=RY+S,$$

$${\mu }_{Z}=3.889;{\sigma }_{Z}=2.59;{\rho }_{{ZX}_{1}}=-0.053;{\rho }_{{ZX}_{2}}=0.017.$$

For model, $${Z}_{NP}=g\left(Y+aS\right)+\left(1-g\right)R\left(Y+aS\right),$$

$${\mu }_{{Z}_{NP}}=52.31;{\sigma }_{{Z}_{NP}}=6.78;{\rho }_{{{Z}_{NP}X}_{1}}=-0.044;{\rho }_{{{Z}_{NP}X}_{2}}=0.140.$$

Table 2 shows the results for MSE estimates of the model given by Diana and Perri^6^ and the proposed model. The resuts are obtained byusing two different sample sizes n = 20 and 38. One can notice that the proposed estimator provides minimum and better results as compared to the other estimators under both models.

**Table 2:** The MSEs of the estimators for real population.

*n*	Estimators	*Z* = *R* × *Y* + *S*		${Z}_{NP}=g\left(Y+aS\right)+\left(1-g\right)R\left(Y+aS\right)$		
		Mean	MSE	Estimators	Mean	MSE
20	${t}_{0}$	5.3726	8.2592	${t}_{NP1}$	73.5542	229.6445
	${t}_{ratio}$	7.9481	4.2433	${t}_{NP2}$	107.2465	78.2292
	${t}_{1D}$	3.4357	3.3778	${t}_{1N}$	47.0329	64.1311
38	${t}_{0}$	5.3279	3.4451	${t}_{NP1}$	73.5636	96.6005
	${t}_{ratio}$	6.4387	2.3176	${t}_{NP2}$	87.7502	96.1805
	${t}_{1D}$	3.4072	1.4090	${t}_{1N}$	47.0429	39.5865

## Simulation study

In this section, we conduct a simulation study to evaluate the performance of the proposed generalized exponential-type estimators by comparing some existing variance estimators.

Population I:

$$\Sigma =\left[\begin{array}{ccc}10& 3& 2.9\\ 3& 2& 1.1\\ 2.9& 1.1& 2\end{array}\right], {\rho }_{x1y}=0.6817, { \rho }_{x2y}=0.6705.$$

Population II:

$$\Sigma =\left[\begin{array}{ccc}6& 3& 2.9\\ 3& 2& 1.1\\ 2.9& 1.1& 2\end{array}\right], {\rho }_{x1y}=0.8706, { \rho }_{x2y}=0.8706.$$

For both populations, we ruminate three different samples of sizes 200, 300, and 500. The variance of *S* i.e. Var (*S*) and variance of *T*, i.e.Var (*T*) choose different values for simulation.

Table 3 provides the privacy protection level of the RRT models discussed in this study, we follow Gupta et al.^31^ unified measure of the estimator and is given by

**Table 3:** Privacy level for two populations.

Model	Population I	Population II
Diana and Perri^6^	10.1472	7.1942
Proposed model	5.6528	4.9476

$$\vartheta =\frac{MSE\left({t}_{i}\right)}{{\Delta }_{j}}\times 100,$$

where *i* = 0*, ratio*, 1*D*, *NP*1, *NP*2, 1; *j* = *D* and *PN*, $MSE({t}_{i})$ is the theoretical MSE of the various estimators and ${\Delta }_{j}$ is the privacy level for Diana and Perri’s^6^ model and the proposed model as discussed in “Privacy levels” section.

Tables 4, 5 and 6 give the MSE and percent relative efficiency (PRE) results for the proposed estimator and existing estimators discussed in this article. The following expression is implied to get the PRE,

$$PRE=\frac{MSE({t}_{0})}{MSE({t}_{i})}\times 100,$$

where i = ratio, gratio, and gep.

**Table 4:** The MSEs and PREs of the estimators for Population I with ${\sigma }_{T}^{2}=0.5$ using Z = YT + S.

*Var(S)*	*N*	Estimators	Mean $\left( {\widehat{\sigma }_{y}^{2} } \right)$	MSE	PRE	$\vartheta$
0.2	200	${t}_{0}$	12.6563	4.1872	100.00	0.4126
		${t}_{ratio}$	12.7547	4.1573	100.72	0.4097
		${t}_{gratio}$	12.6568	4.1013	102.09	0.4042
		${t}_{1D}$	12.0520	3.9767	105.29	0.3919
	500	${t}_{0}$	12.6540	1.2985	100.00	0.1280
		${t}_{ratio}$	12.6910	1.0253	126.65	0.1010
		${t}_{gratio}$	12.6539	1.0065	129.01	0.0980
		${t}_{1D}$	12.0586	0.2382	545.13	0.0235
0.5	200	${t}_{0}$	14.6764	4.1979	100.00	0.4137
		${t}_{ratio}$	14.8676	4.0264	104.26	0.3968
		${t}_{gratio}$	14.6795	3.9598	106.01	0.3902
		${t}_{1D}$	13.0802	2.9901	140.39	0.0976
	500	${t}_{0}$	14.7046	1.4063	100.00	0.1386
		${t}_{ratio}$	14.7376	1.0221	137.59	0.1007
		${t}_{gratio}$	14.7047	0.9945	141.41	0.0980
		${t}_{1D}$	13.0697	0.2428	579.20	0.0239
1	200	${t}_{0}$	13.1945	3.2376	100.00	0.3191
		${t}_{ratio}$	13.3712	3.2163	100.66	0.3170
		${t}_{gratio}$	13.1975	3.1964	101.29	0.3150
		${t}_{1D}$	12.0749	0.9605	337.07	0.0947
	500	${t}_{0}$	13.1984	1.3192	100.00	0.1300
		${t}_{ratio}$	13.2229	0.8057	163.73	0.0794
		${t}_{gratio}$	13.1979	0.7942	166.10	0.0783
		${t}_{1D}$	12.0569	0.2420	545.12	0.0238

**Table 5:** The MSEs and PREs of the estimators for Population II with ${\sigma }_{T}^{2}=0.5$ using Z = YT + S.

Var(S)	N	Estimators	Mean $\left( {\widehat{\sigma }_{y}^{2} } \right)$	MSE	PRE	$\vartheta$
0.2	200	${t}_{0}$	8.1658	1.5367	100.00	0.2136
		${t}_{ratio}$	8.2248	1.4245	107.88	0.1980
		${t}_{gratio}$	8.1710	1.3286	115.66	0.1847
		${t}_{1D}$	8.0373	1.1215	137.02	0.1559
	500	${t}_{0}$	8.1555	0.3809	100.00	0.0529
		${t}_{ratio}$	8.1822	0.3570	106.69	0.0496
		${t}_{gratio}$	8.1582	0.3324	114.59	0.0462
		${t}_{1D}$	8.0517	0.2871	132.67	0.0399
0.5	200	${t}_{0}$	8.9991	2.1105	100.00	0.2934
		${t}_{ratio}$	9.0607	1.8331	115.13	0.2548
		${t}_{gratio}$	9.0032	1.7227	122.51	0.2395
		${t}_{1D}$	8.0269	1.1276	187.17	0.1567
	500	${t}_{0}$	8.9905	0.5165	100.00	0.0718
		${t}_{ratio}$	9.0144	0.4564	113.17	0.0634
		${t}_{gratio}$	8.9923	0.4291	120.37	0.0596
		${t}_{1D}$	8.0471	0.2798	184.60	0.0389
1	200	${t}_{0}$	8.7447	2.7373	100.00	0.3805
		${t}_{ratio}$	8.8042	2.6687	102.57	0.3710
		${t}_{gratio}$	8.7519	2.5569	107.06	0.3554
		${t}_{1D}$	8.0489	1.1238	243.58	0.1562
	500	${t}_{0}$	8.7367	0.6678	100.00	0.0928
		${t}_{ratio}$	8.7636	0.6414	104.12	0.0892
		${t}_{gratio}$	8.7390	0.6144	108.69	0.0854
		${t}_{1D}$	8.0495	0.2794	239.01	0.0388

**Table 6:** The MSEs of the estimators for population I and II with $\sigma_{T}^{2}$ using the proposed model.

Var(S)	*N*	Estimators	Population I			$\vartheta$	Population II			$\vartheta$
			Mean $\left( {\widehat{\sigma }_{y}^{2} } \right)$	MSE	PRE		Mean $\left( {\widehat{\sigma }_{y}^{2} } \right)$	MSE	PRE	
0.2	200	${t}_{NP1}$	12.8756	3.3689	100.00	0.5960	7.9867	1.3649	100.00	0.2759
		${t}_{NP2}$	13.0140	3.5686	94.40	0.6313	8.0386	1.2930	105.56	0.2613
		${t}_{1N}$	11.0777	1.0035	335.71	0.1775	7.0332	1.1664	117.02	0.2358
	500	${t}_{NP1}$	12.8432	0.8166	100.00	0.1445	7.5139	0.3972	100.00	0.0803
		${t}_{NP2}$	12.8931	1.1165	73.14	0.1975	7.5327	0.3666	108.35	0.0741
		${t}_{1N}$	11.0683	0.2599	314.20	0.0460	7.0452	0.2466	161.07	0.0498
0.5	200	${t}_{NP1}$	13.4345	4.4759	100.00	0.7918	8.0543	1.8112	100.00	0.3661
		${t}_{NP2}$	13.5454	5.0172	89.21	0.8876	8.1007	1.5241	118.84	0.3080
		${t}_{1N}$	12.0603	1.0186	439.42	0.1802	7.4002	1.1855	152.78	0.2396
	500	${t}_{NP1}$	13.4065	1.0886	100.00	0.1926	8.0695	0.4521	100.00	0.0914
		${t}_{NP2}$	13.4385	1.2013	90.62	0.2125	8.0792	0.3813	118.57	0.0771
		${t}_{1N}$	12.0664	0.2485	438.07	0.0440	7.0388	0.2986	151.41	0.0604
1	200	${t}_{NP1}$	13.9783	4.5249	100.00	0.8005	7.6827	1.9927	100.00	0.4028
		${t}_{NP2}$	14.0973	4.8623	93.06	0.8602	7.7180	1.6920	117.77	0.3420
		${t}_{1N}$	13.0412	1.3838	326.99	0.2448	7.1079	1.2108	164.58	0.2447
	500	${t}_{NP1}$	13.9681	1.0967	100.00	0.1940	7.7117	0.5027	100.00	0.1016
		${t}_{NP2}$	13.9922	1.3800	79.47	0.2441	7.7162	0.4298	116.96	0.0869
		${t}_{1N}$	13.0686	0.3347	327.67	0.0592	7.0337	0.2945	170.70	0.0595

The results are presented in Tables 4, 5 and 6. The Tables 4 and 5 provides the numerical results of estimators dicussed in “Sampling strategy for scrambled response model” and “The proposed estimator and its class of estimators” sections whereas the Table 6 presented the results of estimators discussed in “The proposed RRT model and estimator-II” section based on proposed model. The values from Tables 4, 5 and 6 confirm that the existing estimators presented by Gupta et al.^28^ are less efficient as compared to the generalized estimator. Also while comparing the proposed model and existing model estimator results in these tables on mayobseve that the proposed model provides more efficient MSE values as compared to the model presented by Diana and Perri^6^. As we can see as the variance of S increases the MSE decreases.

A smaller value of $\vartheta$ is to be preferred. Tables 3, 4 and 5 presents the unified measure along with the PRE of the estimators. It is observed that the proposed generalized estimator using two auxiliary variables efficiently performs either using Diana and Perri’s model or the proposed RRT model. One can notice that the values of $\vartheta$ are smaller for the proposed generalized model.

## Conclusion

This study addressed the estimation of population variance for sensitive study variables using a non-sensitive auxiliary variable. A generalized exponential-type estimator, based on Diana and Perri’s^6^ randomized response model, was introduced and evaluated against estimators proposed by Gupta et al.^28^, as detailed in Tables 4, 5 and 6. The comparative analysis indicated that the proposed estimator consistently demonstrated superior efficiency in variance estimation. Additionally, we introduced a novel generalized scrambled response model and applied it to conventional variance and ratio estimators, along with the proposed estimator. In “An application of the proposed model” section, a real survey-based study was presented, applying the proposed RRT model. The results, obtained under both our novel model and the model presented by Diana and Perri^6^, revealed that the proposed estimator consistently outperformed conventional mean and ratio estimators in minimizing MSE. Notably, as the sample size increased, the efficiency of the estimator further improved. Moreover, a simulation study was conducted, and the findings are summarized in Tables 3, 4, 5 and 6, comparing expected variances, MSE, and the precision (PRE). The results indicated that the generalized proposed estimator under the proposed randomized response model consistently provided the minimum MSE for both populations, outperforming the estimator’s MSE results using Diana and Perri’s^6^ model. This research study contributes valuable insights into variance estimation for sensitive variables. The proposed generalized estimator, underpinned by the innovative scrambled response model, demonstrated robustness, scalability, and superior performance in both real and simulated scenarios. These findings underscore the potential of this approach in advancing the precision and reliability of population variance estimation in sensitive contexts.

## Data Availability

The data set used and/or analysed during the current study available from the corresponding author on reasonable request.
